# Slow-growing broilers are healthier and express more behavioural indicators of positive welfare

**DOI:** 10.1038/s41598-020-72198-x

**Published:** 2020-09-16

**Authors:** Ann C. Rayner, Ruth C. Newberry, Judit Vas, Siobhan Mullan

**Affiliations:** 1 FAI Farms, Northfield Farm, Wytham, OX2 8QJ UK; 2 Division of Food Animal Science, Bristol Veterinary School, University of Bristol, Langford, BS40 5DU UK; 3 Department of Animal and Aquacultural Sciences, Faculty of Biosciences, Norwegian University of Life Sciences, 1432 Ås, Norway

**Keywords:** Animal behaviour, Agricultural genetics

## Abstract

Broiler chicken welfare is under increasing scrutiny due to welfare concerns regarding growth rate and stocking density. This farm-based study explored broiler welfare in four conditions representing commercial systems varying in breed and planned maximum stocking density: (1) Breed A, 30 kg/m^2^; (2) Breed B, 30 kg/m^2^; (3) Breed B, 34 kg/m^2^; (4) Breed C, 34 kg/m^2^. Breeds A and B were ‘slow-growing’ breeds (< 50 g/day), and Breed C was a widely used ‘fast-growing’ breed. Indicators of negative welfare, behavioural indicators of positive welfare and environmental outcomes were assessed. Clear differences between conditions were detected. Birds in Condition 4 experienced the poorest health (highest mortality and post-mortem inspection rejections, poorest walking ability, most hock burn and pododermatitis) and litter quality. These birds also displayed lower levels of behaviours indicative of positive welfare (enrichment bale occupation, qualitative ‘happy/active’ scores, play, ground-scratching) than birds in Conditions 1–3. These findings provide farm-based evidence that significant welfare improvement can be achieved by utilising slow-growing breeds. There are suggested welfare benefits of a slightly lower planned maximum stocking density for Breed B and further health benefits of the slowest-growing breed, although these interventions do not offer the same magnitude of welfare improvement as moving away from fast-growing broilers.

## Introduction

Commercial broiler chicken welfare is receiving increasing scrutiny from the media and Non-Governmental Organisations (NGOs) concerning welfare issues associated with rapid growth and rearing conditions.European and North American NGOs are targeting food companies, requesting that they meet a number of requirements to “best mitigate […] the most pressing welfare concerns relating to broiler production”^1,2^. Two of these requirements are: to “implement a maximum stocking density of 30 kg/m^2^” (6.0lbs/ft^2^ in North America) and to “adopt breeds that demonstrate higher welfare outcomes”. These requirements raise questions about the impact of varying combinations of stocking density and breed on the welfare of broilers under commercial conditions.

Stocking density is the “total live weight of chickens present in a house at the same time per square metre of usable area”^3^. The European Council Broiler Directive (2007/43/EC) sets a maximum stocking density (providing specific requirements are met) of 42 kg/m^2^ whilst the UK codes of practice do not permit stocking densities over 39 kg/m^2^^4^. Most UK retailer standards state a maximum stocking density of 38 kg/m^2^. ‘Higher welfare’ retailer standards may set somewhat more stringent upper limits (e.g. 34 kg/m^2^) or even more stringent limits in line with the NGO ‘Chicken Commitment’ requirements^1,2^. Higher stocking densities typically result in greater economic returns for broiler producers due to increased numbers of animals and, therefore, kg of meat produced per house. To avoid exceeding maximum stocking densities, in practice, the number of chicks placed at the start is calculated from the available floor area of the house and the target final weight of the birds (whilst also allowing for some mortality). For the same final target weight, a higher stocking density would equate to more animals within a given area (animal density). It has been reported that broilers will actively work to avoid higher stocking densities in certain contexts^5^ whilst clustering together in other contexts^6^. Distance travelled or walking bout length decreases with stocking density^6,7^ and broilers have been observed to ‘jostle’ one another^8^ and experience more interruptions to resting periods^9^ at higher densities. In general, there is a trend for reduced health of broilers at higher stocking densities, including poorer walking ability^6,10–12^ and increased footpad dermatitis^11,13,14^.

Global broiler production generally utilises breeds with mean growth rates of > 50 g/day (‘fast-growing broilers’). ‘Slow-growing broilers’ (< 50 g/day) are supplied by traditional breed providers or arms of the major genetics companies. The market for slow-growing breeds is currently a small portion of all broiler production (for the UK this is estimated to be around 11%^15^). Interest in slow-growing broilers is driven by diverse region-specific consumer trends, including animal welfare interest (promoted by NGOs), legislation for age at processing, demands from traditional cuisines and retailer initiatives to create premium products (e.g. ‘Higher welfare’ branded meat products). There are few published direct comparisons of breeds, particularly under commercial production or undertaken within the last 10 years. Given the fast development of broiler genetics previous research may not be reflective of today’s genetics. Pen trials have, however, shown differences in behaviour between breeds. For example, Bokkers and Koene^16^ reported that slow-growing broilers perched, walked and ground-scratched more whereas fast-growing broilers sat, ate and drank more. Further, birds growing at > 41 g/day performed a reduced variety of behaviours when compared to breeds growing at 25–40 g/day and < 24 g/day^17^. Fast-growing breeds have also been reported to have poorer walking ability^18–20^, more foot lesions^16,17,21^, higher mortality, culls and biological indicators of poorer immunity^17^.

Assessments of broiler welfare such as those described above have typically focused on negative welfare outcomes. Recently, there has been an evolution of welfare science to explore positive experiences of animals^22^ recognising that good welfare, a “good life”^23–25^, is not just about negating negative states but also the promotion of positive experiences and emotional states. Positive animal welfare and its assessment emphasises resources that are valued by animals^25^ as well as positive emotions and the natural behaviours animals are motivated to perform^22^.

The aim of this on-farm study was to evaluate the welfare of broilers in four commercially relevant systems with varying combinations of breed (across three breeds selected for different growth rates) and stocking density (planned for 30 vs 34 kg/m^2^ at slaughter age). This study is the first to utilise an extensive suite of specific behavioural measures of positive welfare alongside more traditional negative welfare outcomes and environmental outcomes in a large-scale trial. We predicted that negative welfare outcomes would increase, and positive welfare outcomes would decrease, with increased mean growth rate and stocking density, equivalent to increased productivity of the system. Thus, we expected that the condition that would achieve the best welfare would be that with the slowest growing birds and lowest stocking density.

## Results

### Production information

There was a 14 day difference in production cycle length between Conditions 1 and 4 (Table 1a). This difference in growth rate was already apparent at Production Stage 1, with birds in Condition 4 being 41% heavier than the birds in Condition 1. While final animal densities remained different for the two planned maximum stocking densities, final stocking densities were lower than planned based on a target weight of 2.2 kg.

**Table 1:** (a) Production information and (b) production-related negative welfare outcomes by Condition (Mean ± SE per production cycle).

Variable	Condition 1	Condition 2	Condition 3	Condition 4
**(a) Production information**				
Breed	A	B	B	C
Planned maximum stocking density (kg/m^2^)	30	30	34	34
Age at processing (d)	49	45	45	35
Production cycles (n)	4	4	4	4
Chicks placed at start (n)	11,858 ± 58	11,830 ± 50	13,450 ± 27	13,563 ± 175
**Growth rate (g/day)**				
Production Stage 1 (14 d)	24.61 ± 0.77	25.21 ± 0.51	24.09 ± 0.67	34.86 ± 1.94
Production Stage 2 (28 d)	34.59 ± 6.13	36.98 ± 0.41	37.39 ± 0.19	51.18 ± 1.82
Production Stage 3 (3 d before processing)	43.99 ± 1.28	45.67 ± 0.59	44.99 ± 0.56	56.31 ± 1.66
Final growth rate at processing	44.26 ± 1.60	47.04 ± 0.75	46.91 ± 1.13	58.46 ± 1.34
**Mean weight (kg)**				
Production Stage 1 (14 d)	0.34 ± 0.01	0.35 ± 0.01	0.34 ± 0.01	0.49 ± 0.03
Production Stage 2 (28 d)	0.97 ± 0.02	1.04 ± 0.01	1.05 ± 0.01	1.45 ± 0.04
Production Stage 3 (3 d before processing)	2.02 ± 0.06	1.92 ± 0.02	1.89 ± 0.02	1.80 ± 0.05
Weight at processing	2.17 ± 0.08	2.12 ± 0.03	2.11 ± 0.05	2.05 ± 0.05
**Animal density (birds/m**^2^)				
Animal density at placement	13.42 ± 0.08	13.39 ± 0.07	15.23 ± 0.04	15.30 ± 0.20
Production Stage 1 (14 d)	13.09 ± 0.27	13.18 ± 0.08	15.06 ± 0.08	14.84 ± 0.15
Production Stage 2 (28 d)	12.98 ± 0.30	13.07 ± 0.10	14.96 ± 0.10	14.57 ± 0.13
Production Stage 3 (3 d before processing)	12.93 ± 0.31	13.00 ± 0.12	14.89 ± 0.12	14.49 ± 0.12
Animal density at processing	12.91 ± 0.31	12.98 ± 0.12	14.86 ± 0.12	14.41 ± 0.13
Final stocking density (kg/m^2^)	28.05 ± 1.47	27.48 ± 0.57	31.38 ± 0.93	29.47 ± 0.65
**(b) Production-related negative welfare outcomes**				
7d Mortality (%)*				
Leg Culls	0.15 ± 0.04	0.28 ± 0.06	0.21 ± 0.06	0.30 ± 0.05
Other Culls	0.13 ± 0.01	0.22 ± 0.04	0.18 ± 0.06	0.33 ± 0.08
Dead	0.48 ± 0.08	0.77 ± 0.12	0.57 ± 0.11	1.45 ± 0.28
7d Total	0.76 ± 0.12	1.27 ± 0.19	0.96 ± 0.22	2.08 ± 0.29
Total Mortality (%)*				
Leg Culls	0.49 ± 0.12	0.78 ± 0.21	0.62 ± 0.17	2.09 ± 0.39
Other Culls	0.41 ± 0.04	0.71 ± 0.18	0.60 ± 0.19	1.03 ± 0.03
Dead	1.21 ± 0.14	1.71 ± 0.23	1.37 ± 0.21	3.11 ± 0.49
Total	2.10 ± 0.30	3.20 ± 0.54	2.58 ± 0.56	6.23 ± 0.62
**Processing welfare outcomes (%)**				
Catching and transport time (hr:min)	3:26 ± 0:19	4:09 ± 0:13	3:35 ± 0:20	3:36 ± 0:17
Dead on Arrival	0.02 ± 0.01	0.03 ± 0.03	0.02 ± 0.01	0.04 ± 0.02
Pre-processing Culls	0.02 ± 0.01	0.02 ± 0.01	0.02 ± 0.01	0.14 ± 0.06
Total Post-mortem Inspection Rejections	0.14 ± 0.03	0.45 ± 0.15	0.60 ± 0.15	1.34 ± 0.37

*n = 4 production cycles for Conditions 2, 3 and 4; n = 3 for Condition 1.

### Negative welfare outcomes

#### Mortality

Condition 4 resulted in the numerically highest 7d and Total Mortality (Table 1b; Fig. S1, Supplementary Information). Production Cycle 2 of Condition 1 experienced high 7d Mortality. Because it occurred only in one production cycle, this mortality was unlikely to have been related specifically to Condition 1 and so mortality data from this production cycle were excluded in Table 1. When including the Production Cycle 2 mortality figures in the mean score (± SE), Condition 1 had 2.27 ± 1.52% 7d Mortality and 4.00 ± 1.91% Total Mortality.

#### Processing welfare outcomes

**Figure 1:**
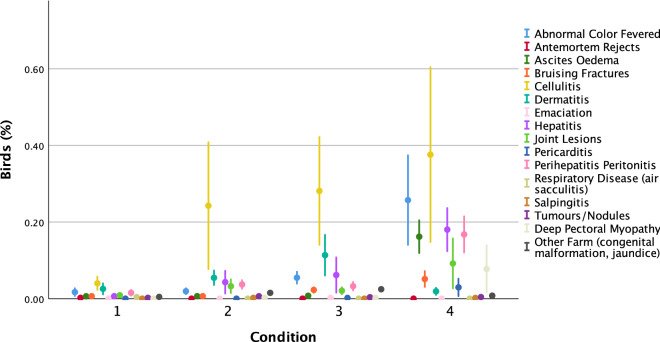
Mean percentage (± SE) Post-mortem Inspection Rejection reasons across Condition (n = 4 production cycles). Figure created in IBM SPSS version 25 (https://www.ibm.com/analytics/spss-statistics-software).

All conditions had a similar percentage of birds Dead on Arrival at the processor but Condition 4 had a greater percentage of Pre-processing Culls (Table 1b). A stepwise increase in Total Post-mortem Inspection Rejections was observed from Condition 1–4. Condition 4 had 9.6 times more rejections than Condition 1 as well as a greater variety of reasons for rejection (Fig. 1).

#### Avoidance distance test

Median (IQR) percentages of birds within arm’s reach were 14.29 (5.00), 4.96 (2.00), 0.00 (1.00) and 0.00 (1.00) for Conditions 1–4, respectively (χ_3_^2^ = 65.32, p < 0.001; Fig. S2). Post-hoc analysis revealed differences between all conditions except between Conditions 3 and 4.

#### Gait score

**Figure 2:**
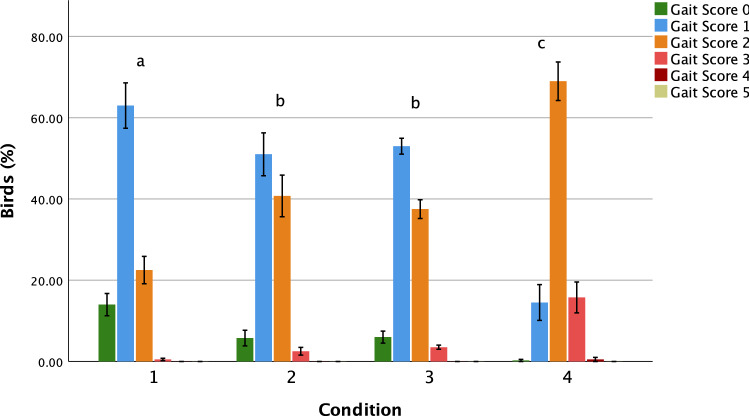
Mean (± SE) percentage of birds with each Gait Score (ranging from 0, walks with ease, to 5, unable to walk) by Condition (n = 100 birds per production cycle 2d before processing, across four production cycles). Different letters indicate differences in Gait Score distribution between conditions as identified by pairwise comparisons using Dunn’s^52^ procedure (p < 0.0083). Figure created in IBM SPSS version 25 (https://www.ibm.com/analytics/spss-statistics-software).

Conditions 1–3 had 0.5, 2.5 and 3.5% of birds with Gait Score 3 or greater, compared to 16.25% of birds in Condition 4 (Fig. 2). Mean (± SE) Gait Scores for Conditions 1–4 were 1.10 ± 0.03, 1.42 ± 0.03, 1.39 ± 0.03 and 2.02 ± 0.03%, respectively (χ_3_^2^ = 368.73, p < 0.001; n = 400 birds per Condition), with pairwise differences between all conditions except between Conditions 2 and 3 (Fig. 2).

#### Hock burn and pododermatitis

**Figure 3:**
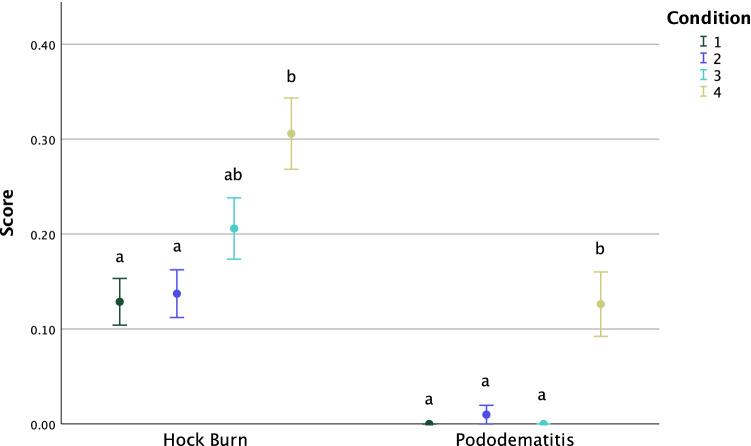
Mean (± SE) Hock Burn and Pododermatitis score (ranging from 0, no evidence of lesion, to 4, severe lesions) by Condition (n = 202, n = 204, n = 204, n = 206 birds scored in Conditions 1–4, respectively) 2d before processing of Production Cycles 3 and 4. Different letters indicate differences in score distribution within each variable, between conditions as identified by pairwise comparisons of medians using Dunn’s^52^ procedure (p < 0.0083). Figure created in IBM SPSS version 25 (https://www.ibm.com/analytics/spss-statistics-software).

In the final two production cycles, Conditions 1–4 had 12.38, 13.24, 18.14 and 26.70% of birds with signs of Hock Burn (score 1 or 2), respectively (χ_3_^2^ = 19.08, p < 0.001). Post hoc analysis revealed that the scores in Conditions 1 and 2 differed from those in Condition 4 (Fig. 3). No birds in Conditions 1 and 3 had evidence of Pododermatitis whereas, in Conditions 2 and 4, 0.5 and 7.28% of birds had signs of Pododermatitis (scores 1–3), respectively (χ_3_^2^ = 40.66, p < 0.001). Post hoc analysis revealed differences between Conditions 1, 2 and 3 vs Condition 4 (Fig. 3).

### Positive welfare outcomes

#### Bales occupied

**Figure 4:**
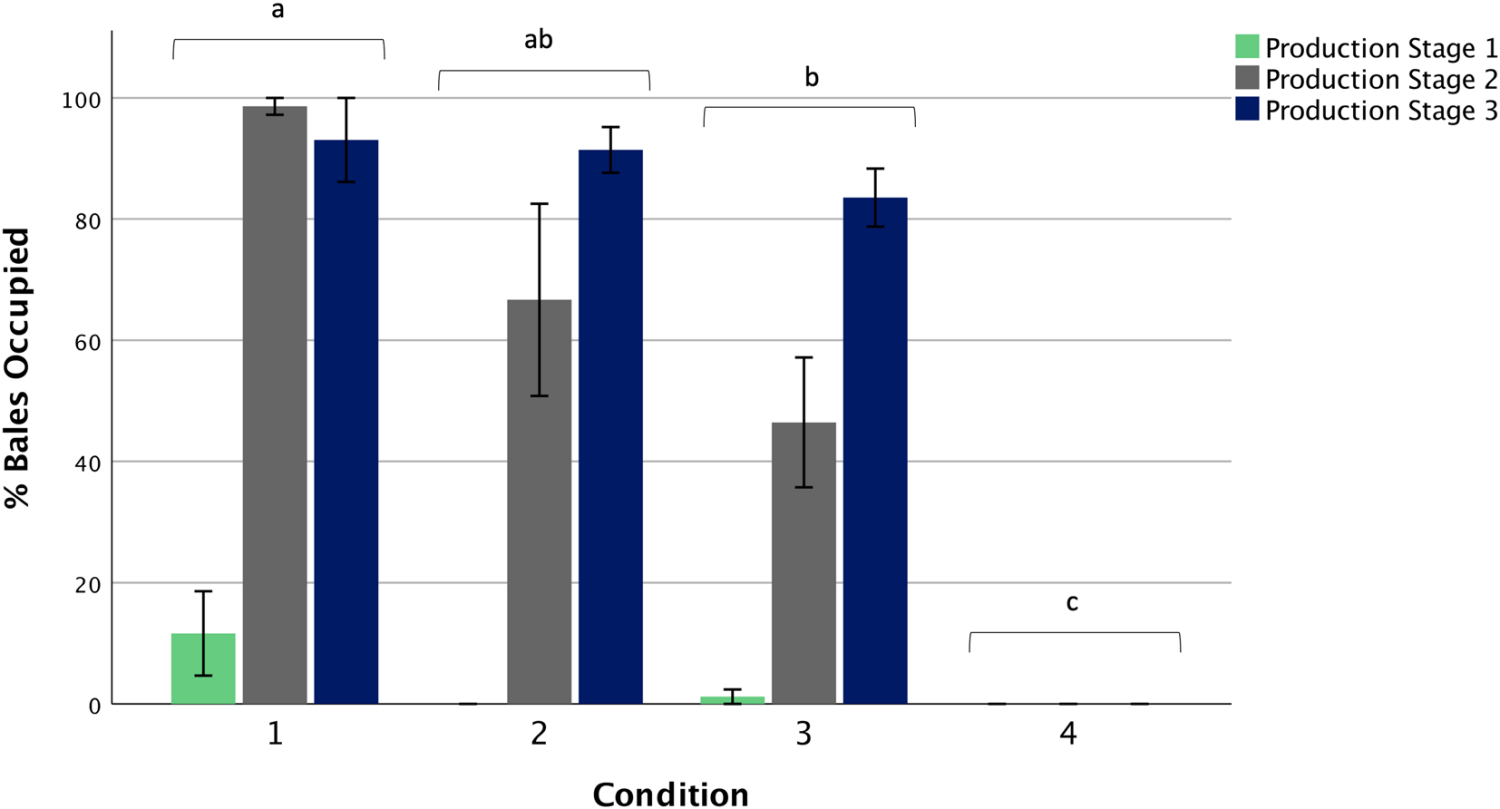
Mean percentage (± SE) of enrichment Bales Occupied by Condition at each Production Stage (n = 4). Different letters indicate differences in Bales Occupied from post-hoc pairwise comparisons of estimated marginal means for Condition utilising a Bonferroni correction (p < 0.0083). Figure created in IBM SPSS version 25 (https://www.ibm.com/analytics/spss-statistics-software) and Microsoft PowerPoint for Mac 2020 version 16.40 (www.microsoft.com).

Mean (± SE) percentage Bales Occupied at Production Stage 3 was 93.06 ± 6.94, 91.42 ± 3.78, 83.53 ± 4.79 and 0.00 ± 0.00 for Conditions 1 to 4, respectively (Fig. 4). There was an interaction between Condition and Production Stage (F_6,26_ = 13.50, p < 0.001, partialŋ^2^ = 0.771). There was also a main effect of Condition (F_3,12_ = 69.71, p < 0.001, partialŋ^2^ = 0.946) and Production Stage (F_2,24_ = 100.30, p < 0.001, partialŋ^2^ = 0.893) on Bales Occupied. Pairwise comparisons revealed a difference between Conditions 1 and 3 (p = 0.002), between Condition 4 and all other conditions (p < 0.001), and between Production Stage 1 vs 2 (p < 0.001) and 1 vs 3 (p < 0.001). Bales Occupied did not differ between Production Stages 2 and 3 (p = 0.084).

#### Qualitative behaviour assessment

From principal component (PC) analysis of 48 assessments, two main PCs (PC1, PC2) were identified by visual inspection for the point of deflection in the Scree plot. PC1 and PC2 together explained 54.10% of the variance (39.18 and 14.90%, respectively). PC1 ranged from ‘Happy/Active’ to ‘Flat/Stressed’ and PC2 ranged from ‘Calm’ to ‘Flighty/Alert’ (Table S3).

**Figure 5:**
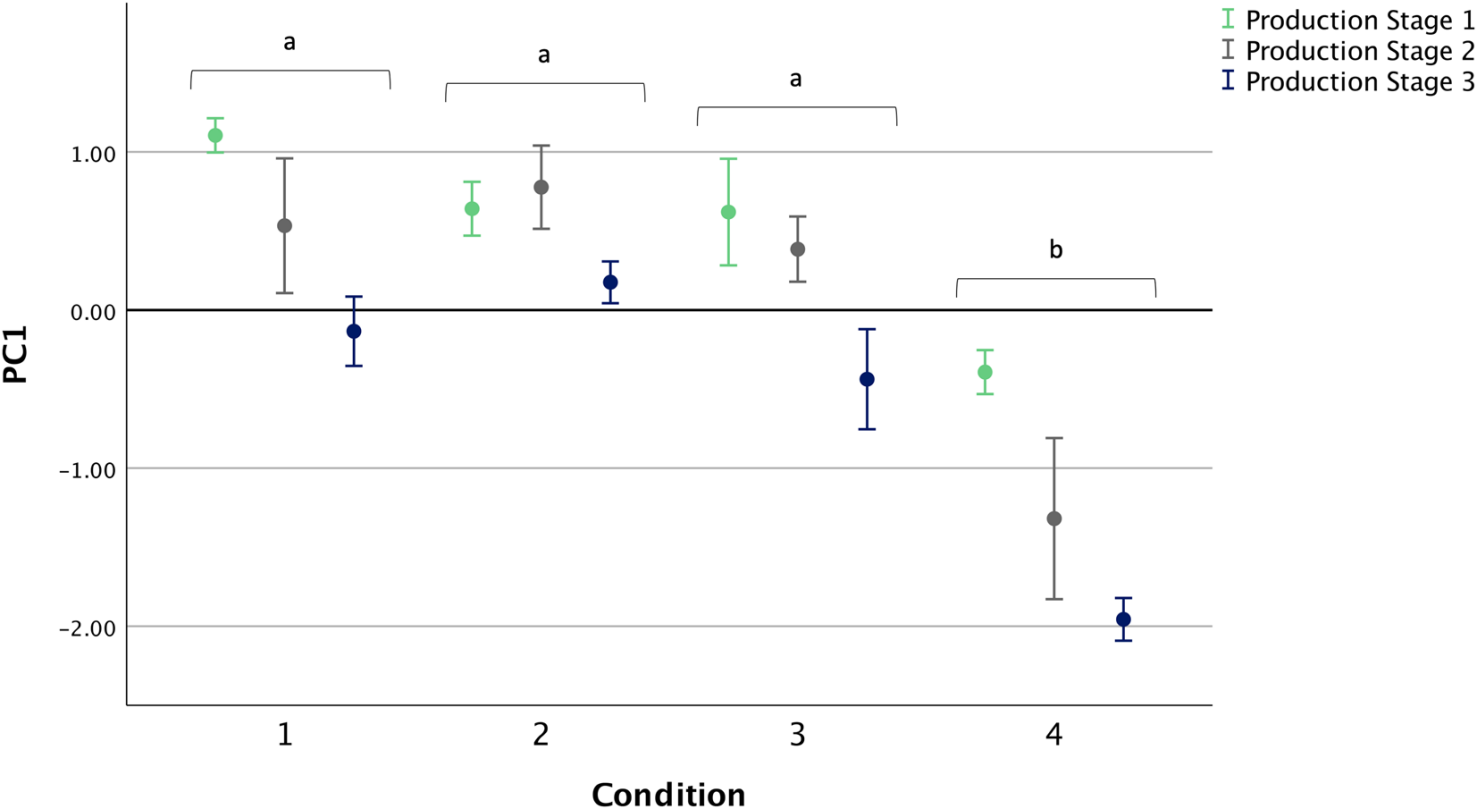
Mean (± SE) Qualitative Behaviour Assessment principal component (PC) scores for PC1, which ranged from ‘Happy/Active’ (high scores) to ‘Flat/Stressed’ (low scores), by Condition at each Production Stage (n = 4). Different letters indicate differences in scores from post-hoc pairwise comparisons of estimated marginal means for Condition utilising a Bonferroni correction (p < 0.0083). Figure created in IBM SPSS version 25 (https://www.ibm.com/analytics/spss-statistics-software) and Microsoft PowerPoint for Mac 2020 version 16.40 (www.microsoft.com).

There was no interaction between Condition and Production Stage in PC1 (F_6,24_ = 0.765, p = 0.604, partialŋ^2^ = 0.161; Fig. 5). However, PC1 scores differed between conditions (F_3,12_ = 50.73, p < 0.001, partialŋ^2^ = 0.927; Fig. 5), with pairwise comparisons revealing differences between Condition 4 and all other Conditions (p < 0.001). Birds in Condition 4 were scored as being more ‘Flat/Stressed’. PC1 scores were also associated with Production Stage (F_2,24_ = 12.80, p < 0.001, partialŋ^2^ = 0.516), with pairwise comparisons showing a reduction in score (from ‘Happy/Active’ towards ‘Flat/Stressed’) between Production Stage 1 and 3 (p < 0.001).

For PC2, there was no interaction between Condition and Production Stage (F_6,24_ = 0.329, p = 0.915, partialŋ^2^ = 0.076), and no main effect of Condition (F_3,12_ = 0.231, p = 0.873, partialŋ^2^ = 0.055) or Production Stage (F_2,24_ = 0.477, p = 0.626, partialŋ^2^ = 0.038). The mean (± SE) PC2 scores for Conditions 1 to 4 were − 0.25 ± 0.31, 0.16 ± 0.30, − 0.01 ± 0.21 and 0.10 ± 0.33 respectively.

#### Positive behaviour observations

**Figure 6:**
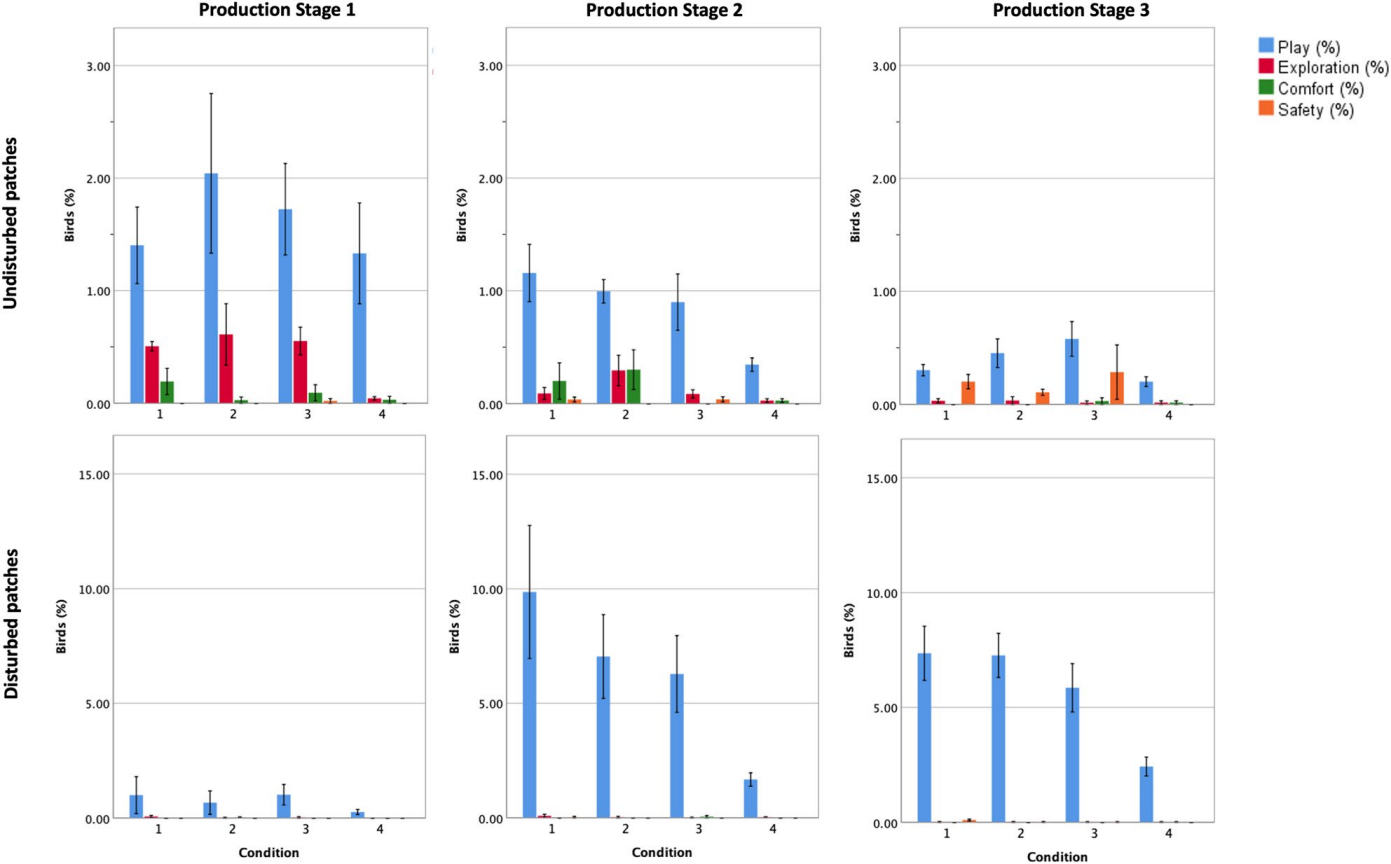
Percentage (± 2 SE) of birds (of total expected) displaying each positive behaviour category in Disturbed and Undisturbed patches by Condition at each Production Stage (n = 4). See Table S2 for behaviour definitions, and Table S4 for significance of differences associated with Disturbance and Production Stage. Figure created in IBM SPSS version 25 (https://www.ibm.com/analytics/spss-statistics-software) and Microsoft PowerPoint for Mac 2020 version 16.40 (www.microsoft.com).

Positive behaviour rates varied between conditions depending on whether or not patches were disturbed (Fig. 6; Table S4). Condition was a significant predictor of Play behaviours in Disturbed patches, of Exploratory behaviour in Undisturbed patches and of Any Positive behaviour in Disturbed patches. Where Play behaviours were relatively uncommon in Undisturbed patches, they were seen frequently in Conditions 1–3 (OR (95% CI): 4.09 (1.79–9.34), 3.20 (1.40–7.29), 3.27 (1.43–7.48), respectively) compared to Condition 4 in Disturbed patches (p = 0.004). Exploratory behaviour (represented by ground-scratching) was seen more often in Conditions 1–3 that in Condition 4 in Undisturbed patches (OR (95% CI): 5.95 (1.60–22.09), 10.16 (2.80–36.75), 5.63 (1.53–20.74), respectively; p = 0.006) but did not vary between conditions in Disturbed patches. Odds of observing Any Positive Behaviour followed the same pattern as Play, being 4.17 (1.82–9.51), 3.24 (1.42–7.37) and 3.28 (1.44–7.50) times higher for Conditions 1–3, respectively, compared to Condition 4 (p = 0.003) in Disturbed patches but not different in Undisturbed patches.

Production Stage was a predictor of positive behaviour rates (Fig. 6; Table S4). In Undisturbed patches, increased odds of observation of Play, Exploratory and Comfort behaviours were observed in Production Stage 1 and 2 compared to Production Stage 3 (OR (95% CI) Play: 4.48 (2.15–9.36), 2.19 (1.04–4.61) respectively, p < 0.001; Exploratory behaviour: 18.06 (5.66–57.59), 4.38(1.33–14.47) respectively, p < 0.001; Comfort behaviour: 6.45 (1.55–26.82), 8.67 (2.09–35.81) respectively, p = 0.011). For Any Positive Behaviour in Undisturbed patches, only Production Stage 1 had increased odds of observation compared to Production Stage 3 (OR (95% CI) 4.05 (1.96–8.39), p < 0.001). In Disturbed patches, odds of observing Play (OR (95% CI) 0.12 (0.06–0.25); p = 0.001) and Any Positive Behaviour (OR (95% CI) 0.13 (0.06–0.26); p < 0.001) were lower in Production Stage 1 than in Production Stage 3.

When exploring associations between stocking density and positive behaviours in Breed B (Condition 2 vs 3), there was no difference in the odds of observing any of the positive behaviours in Undisturbed or Disturbed patches (p > 0.05). Furthermore, when comparing the two slow-growing breeds (A vs B) stocked at similar planned stocking densities (Conditions 1 vs 2), no differences in display of positive behaviours were detected in either Undisturbed or Disturbed patches (p > 0.05).

### Environmental outcomes

#### Litter quality

Litter quality was better in Conditions 1–3, with mean (± SE) litter scores of 0.29 ± 0.03, 0.21 ± 0.02 and 0.46 ± 0.10, respectively, compared to 2.19 ± 0.09 in Condition 4 (n = 4 per condition).

#### Ammonia

Ammonia levels were variable across the four conditions, with mean (± SE) ammonia concentrations of 6.05 ± 0.89, 9.65 ± 0.80, 10.88 ± 0.91 and 7.13 ± 0.48 ppm, respectively (n = 4 per condition).

## Discussion

This is the first study to utilise a comprehensive suite of measures to specifically identify the differences in negative welfare outcomes and positive behavioural outcomes across four commercial broiler systems of varying planned maximum stocking density and breeds. Clear differences in outcomes can be observed particularly between Condition 4 (standard fast-growing breed, highest planned maximum stocking density) and all other conditions. Condition 4 birds experienced the poorest health as indicated by levels of 7d and Total Mortality, Post-mortem Inspection Rejections at processing, Gait Score, Hock Burn and Pododermatitis. In addition, birds in Condition 4 had poorer positive welfare outcomes (Bales Occupied, qualitative ‘Happy/Active’ scores, and Play and Exploratory behaviours) than birds in the other conditions, and poorer Litter Quality. In Breed B, the somewhat higher planned stocking density and resulting higher animal density of Condition 3 vs 2 was associated with a higher Avoidance Distance to the observer. Comparing the two slow-growing breeds (A and B) at similar stocking density, Breed A (Condition 1) had better health for some measures compared to Breed B (Condition 2), though no differences in positive behaviour rates were detected.

Condition 1 had the lowest Post-mortem Inspection Rejections and range of reasons for rejection. This suggests that Breed A was the most resilient and that resilience was affected by breed and growth rate. This is supported by van der Most et al.’s^26^ findings of compromised immune function when selecting for growth rate in poultry.

In addition to having the highest Gait Scores, birds in Condition 4 showed greater avoidance of humans than birds in Conditions 1 and 2, suggesting that they were more fearful or less inquisitive. Previous application of the Avoidance Distance Test as an indicator of welfare in broiler chickens has shown conflicting results. Vasdal et al.^27^ found that flocks of fast-growing broilers with higher gait scores moved away from the observer less, possibly because they were less able or motivated to do so. In the current study, we consider that it was a combination of higher gait scores, reduced motivation and increased fearfulness that meant that the birds’ did not return towards the observer. It should also be considered that birds in Condition 4 were the youngest at the time of assessment, which may have contributed to a more fearful response. Vasdal et al.^27^ found no effect of stocking density on human avoidance, and Tuyttens et al.^28^ observed higher avoidance distances in flocks kept at lower stocking densities. These findings contrast with our observation that Condition 3 birds avoided the observer more than those in Condition 2 (assessed at the same age). Differences in the animal densities may have contributed to these different results, possibly influenced by unrest resulting from increased disturbances at higher densities^6,8^.

Leg problems are a key welfare and production concern. Walking difficulties (lameness) can be associated with both non-infectious and infectious skeletal disorders. Lameness indicates pain^29^ that results in reduced mobility^30^ and access to resources. Behavioural observations have shown that lame birds spend less time standing, running, preening and dustbathing and more time sitting^31,32^. In the current study, birds in Condition 4 had the highest Gait Scores, consistent with previous work showing poorer gaits in fast compared to slow-growing breeds. For example, Kestin et al.^19^ found that slow-growing Hubbard strains had lower mean gait scores than fast-growing genotypes (Ross 308, 508) when reared on the same feeding regime.

Condition 4 birds were found to have the poorest footpad and hock health compared to the other conditions. Broilers that are less active tend to spend a greater proportion of their time sitting in contact with the litter^33^. The higher levels of Hock Burn and Pododermatitis found in Condition 4 birds are consistent with lower activity as indicated by lower Bales Occupied and Play and Exploratory behaviour results. Furthermore, Condition 4 had the poorest Litter Quality, meaning that these inactive birds not only sat for longer but they were also in contact with poorer litter, increasing the likelihood of hock burn^34^ and pododermatitis^35^.

To date, there has been limited use of Qualitative Behaviour Assessment (QBA) in broiler flocks. Where QBA has been applied, there have been mixed results^36^. We found two components. Condition 4 birds were more likely to be ‘Stressed/Flat’ and less ‘Happy/Active’ (Component 1) than birds in the other conditions. Muri et al.^37^ also found two components in commercial fast-growing broilers, explaining a higher percentage of the variance (70%) compared to the 54% found here, but few associations with other welfare outcomes. The authors considered that the homogeneity of the broiler system resulted in limited variation to detect associations and that QBA is perhaps more appropriate for larger animals in smaller groups, allowing for easier observation of behavioural expression. In the current study, the demeanour of birds in Condition 4 (fast-growing birds) was sufficiently different to be detected by QBA. Condition 4 birds were more likely to be scored with negatively valanced, low arousal terms (‘Stressed/Flat’) compared to the other conditions (slow-growing birds).

We assessed the odds of observing several specific behaviours considered indicative of positive welfare. These behaviours were selected based on being easy to recognise from a distance, infrequent enough to be counted and recorded quickly, and expressive of positive qualities such as joyfulness, curiosity, self-care and agency. Condition 4 birds ground-scratched less (representing Exploratory behaviour) when undisturbed, and Played less and performed Any Positive Behaviour less following disturbance, compared to birds in the other three conditions. Foraging involves exploring the environment by scratching and pecking at the substrate^38^ followed by consumption of edible discoveries. Chickens are highly motivated to explore for food despite provision of an ad libitum diet. Foraging is considered a ‘behavioural need’ of chickens^39^, with chickens preferring to obtain some of their diet by working for it^40^. Foraging requires a loose friable substrate. Condition 4 had the poorest litter quality, potentially reducing opportunities and motivation for foraging. Play behaviour was stimulated by disturbance, as it was less often observed in Undisturbed patches. However, even when space opened up in Disturbed patches, birds in Condition 4 (Breed C) had lower odds of expressing Play behaviours than birds in the other conditions, including Condition 3 (Breed B) birds kept a similar planned maximum stocking density. The Condition 4 birds also tended to engage less in vertical wing shaking (representing Comfort behaviour) when undisturbed (p = 0.099) and had no observations of perching on wires (representing Safety behaviour; too rare for statistical assessment). Given that the Condition 4 birds had poorer Gait, and Pododermatitis scores, higher 7d and Total Mortality and higher Post-mortem Inspection Rejections than birds in the other conditions, we suggest that poor physical health was a key contributor to their relatively low display of positive behaviours.

In the Undisturbed patches, a reduction in Play, Exploration, Comfort behaviour and Any Positive Behaviour counts was observed from the first to last Production Stages. Lower Play in the final production stage is consistent with Vasdal et al.’s^41^ finding of less play at 30 days compared to 16 days, and the observed reduction in Exploratory behaviour with increasing age is consistent with Bizeray et al.^38^. For the Disturbed patches, less Play was recorded in Production Stage 1 than when the birds were older. It is possible that these behaviours were not fully captured by our methodology, as the birds moved away from the observer and played in other space that was readily available in Production Stage 1 due to the birds’ small size. Play can occur as a rebound following deprivation of an environmental resource (such as space)^42^. At Production Stages 2 and 3, the creation of space in the observer’s path of movement may have stimulated a rebound effect, resulting in an increase in odds of observing Play behaviours for these Production Stages. Similarly, Baxter et al.^43^ reported more observations of play-like behaviours following displacement by an observer at 4 and 5 weeks than at 3 weeks of age, consistent with our findings from Disturbed patches.

In the current study we assessed both disturbed and undisturbed patches. Whilst a greater variety of behavioural categories was observed in undisturbed patches, the frequency of observation of play behaviours was much lower than in disturbed patches. In order to gain a clearer picture of the nuanced positive behaviours of broilers future research should focus on undisturbed patches, ideally automating the recognition of these relatively rare behaviours. Observations of disturbed patches may be beneficial for auditing purposes, to provide a discussion point for auditors and farmers such that the promotion of positive experiences is incorporated into expected standards of commercial broiler welfare.

Wetter litter has been associated with poorer broiler health^8^. Conditions 1 and 2 had the best Litter Quality while the highest litter scores were observed in Condition 4 (mean score 2.19, where a score of 2 indicated “leaves imprint of foot and will form a ball if compacted, but ball does not stay together well”). Condition 3′s mean score was lower than Condition 4′s but more variable than Condition 1 and 2′s mean score. Poorer quality litter contains more moisture. High stocking densities put pressure on the litter due to increased faeces excreted within a given area. The somewhat higher densities in Condition 3 and 4, would have resulted in higher faeces and litter moisture per unit of floor space^14^. In addition, the more rapid growth of the Condition 4 birds would have resulted in more rapid build-up of faeces in the litter, explaining the higher litter scores in that Condition despite having a similar planned maximum stocking density to Condition 3 as well as a shorter production duration. Ammonia in poultry houses has deleterious human, bird and environmental effects^44^. However, ammonia concentration at the end of the growth period was similar across conditions and did not exceed recommended levels of < 20 ppm^45^ despite the longer production duration of the birds in Conditions 1–3 or higher litter scores in Condition 4. A more detailed study exploring total emissions over the entire production cycle is required to assess the wider environmental impact of higher welfare systems.

Although stocking density is often referred to when considering the welfare of broilers, higher stocking densities are ultimately achieved by placing more chicks in a given area (when producing birds to the same final weight). Final stocking densities are influenced by the growth rate of the birds which in turn is sensitive to a multitude of variables (e.g. pathologies, feed quality, house temperature profiles, management etc.) while final animal densities are less sensitive to these variables as they are only influenced by mortality. This is reflected in the results in this study. While final stocking densities did not vary greatly across conditions, the difference in animal densities did remain consistent with the planned maximum stocking densities to the point of processing. One could hypothesise that it is the animal density that is more likely to impact the welfare of the individual birds due to competition for resources and behavioural disruption, rather than the space available due to the size of the birds. However, for Breed B, we observed no differences between Conditions 2 and 3 (which differed in animal density), in most of the Negative Welfare Outcomes including Gait Score (in agreement with Bailie et al. considering stocking density^11^). Similarly, a lack of association between animal density and Bales Occupied or positive behaviour observations suggests that opportunities to perform behaviours important for positive welfare were no more compromised at the higher animal density. However, we cannot rule out that the lower animal density of Breed B in Condition 2 was also sufficient to restrict behaviour as the birds grew. For example, Comfort behaviour (dustbathing, as measured by vertical wing shakes) when Undisturbed declined across the Production Stages, whereas in studies where birds were stocked at lower stocking densities, no reduction in dustbathing was observed as birds aged^32,46^. The difference in the Avoidance Distance between Conditions 2 and 3 is perhaps reflective of greater fearfulness at the higher animal density. Use of a slightly higher planned maximum stocking density may help to partially mitigate the loss of productivity from moving to a slow-growing breed in commercial systems.

At similar planned maximum stocking and animal densities, birds in Condition 1 (Breed A) were healthier than those in Condition 2 (Breed B) as indicated by lower 7-day and Total Mortality (when excluding the production cycle that experienced high early mortality), lower Post-mortem Inspection Rejections, and better Gait Scores. A lack of behavioural differences between these conditions suggests that Breed B’s ability to perform specific positive behaviours was not impaired by their slightly faster growth rate.

Broiler producers manage their flocks to achieve the best possible results regardless of the specific marketing scheme demands. In this study, although breed and planned maximum stocking density varied, the producer was continuously monitoring and adapting the management of each house to optimise outcomes. This varies from a controlled experimental study in which specific factors are varied and all other factors held constant. Both approaches are valuable in providing different information, the former about outcomes under real-life conditions (emphasising external validity) and the latter about the impact of individual factors under a single set of controlled conditions (emphasising internal validity). The latter typically involves different management practices to those found commercially such as use of smaller flocks and pen sizes, as well as differences in ventilation patterns, litter management, feeding and watering practices, labour routines, biosecurity between experimental units and veterinary practices. Here, we used the former approach, showing how commercial marketing scheme demands for specific product attributes, in this case, use of slower-growing breeds and lower planned maximum stocking density, were associated with broiler welfare outcomes on one commercial farm. By limiting the study to one farm, we were able to control potentially influential environmental variables (stockperson, house size and equipment, processor, processing dates, seasonal and regional conditions), allowing for detection of differences between conditions in a smaller number of production cycles than would have been required if collecting data across multiple farms and companies. Despite this, the small replicate number and variation within variables contraindicated statistical tests on the production information; further replicates would overcome this. As in the case of results from a single research facility, caution is needed in applying our on-farm results more generally.

## Conclusion

In this study, we compared four conditions simulating four possible marketing schemes for broiler chickens varying in growth rate (varying across three breeds) and planned maximum stocking density (higher vs lower). We predicted that a wide range of negative and positive welfare measures would, overall, indicate the highest welfare in the slowest-growing breed (Breed A) kept at the lower planned maximum stocking density (Condition 1) and the lowest welfare in the fastest-growing strain (Breed C) kept at the higher planned maximum stocking density (Condition 4), with Conditions 2 (slower-growing Breed B at the lower planned maximum stocking density) and 3 (slower-growing Breed B at the higher planned maximum stocking density) having intermediate results. The differences between Conditions 1 to 3 were subtle. Nevertheless, the overall results suggest that, on average, chickens experienced better welfare in Condition 1 than in Conditions 2 and 3 (based on lower 7d and Total Mortality (when excluding one production cycle), fewer Post-mortem Inspection Rejections and variety of reasons for rejection and lower Gait Scores). Differences in welfare between Condition 2 and 3 were less prominent with only a difference in Avoidance Distance Test results. The clearest pattern of findings was for higher welfare in Conditions 1 to 3 when compared with Condition 4 (lower 7d and Total Mortality, Post-mortem Inspection Rejections, Gait Score, Hock Burn and Pododermatitis scores, more Bales Occupied, ‘better’ PC1 Qualitative Behaviour Assessments, higher Play behaviours in Disturbed patches and Exploratory behaviour in Undisturbed Patches). These findings, obtained from an observational study under practical commercial conditions, indicate that the most significant overall welfare improvement can be achieved utilising a slow-growing breed compared to standard fast-growing breeds. There were suggested benefits of utilising a slightly lower planned maximum stocking density and further health benefits in systems utilising the slowest growing genotype. However, these benefits did not give welfare benefits of the same magnitude as could be realised by moving away from the fast-growing broilers (Condition 4, Breed C).

## Material and methods

### Conditions

Birds were kept in four conditions varying in breed and planned maximum stocking density representing commercial production systems:

Condition 1: Breed A (expected growth rate 45 g/day); planned maximum stocking density 30 kg/m^2^.

Condition 2: Breed B (expected growth rate 49 g/day); planned maximum stocking density 30 kg/m^2^.

Condition 3: Breed B (expected growth rate 49 g/day); planned maximum stocking density 34 kg/m^2^.

Condition 4: Breed C (expected growth rate 63 g/day); planned maximum stocking density 34 kg/m^2^.

Breeds A and B were ‘slow-growing’ breeds (< 50 g/day) and Breed C was a standard ‘fast-growing’ (> 50 g/day) breed. Commercial sensitivities preclude identifying breed names. The commercial nature of the study meant that the combinations of breed/planned maximum stocking density available for investigation were limited.

The four conditions were pseudo-randomly allocated across four houses over four production cycles. Full random allocation was not possible as Condition 4 had to be housed in one of two houses due to feed bin requirements. Chicks were placed as hatched and placement was staggered with the intention that birds within a production cycle would reach a targeted final weight of 2.2 kg on the same processing date.

### Housing and management

**Figure 7:**
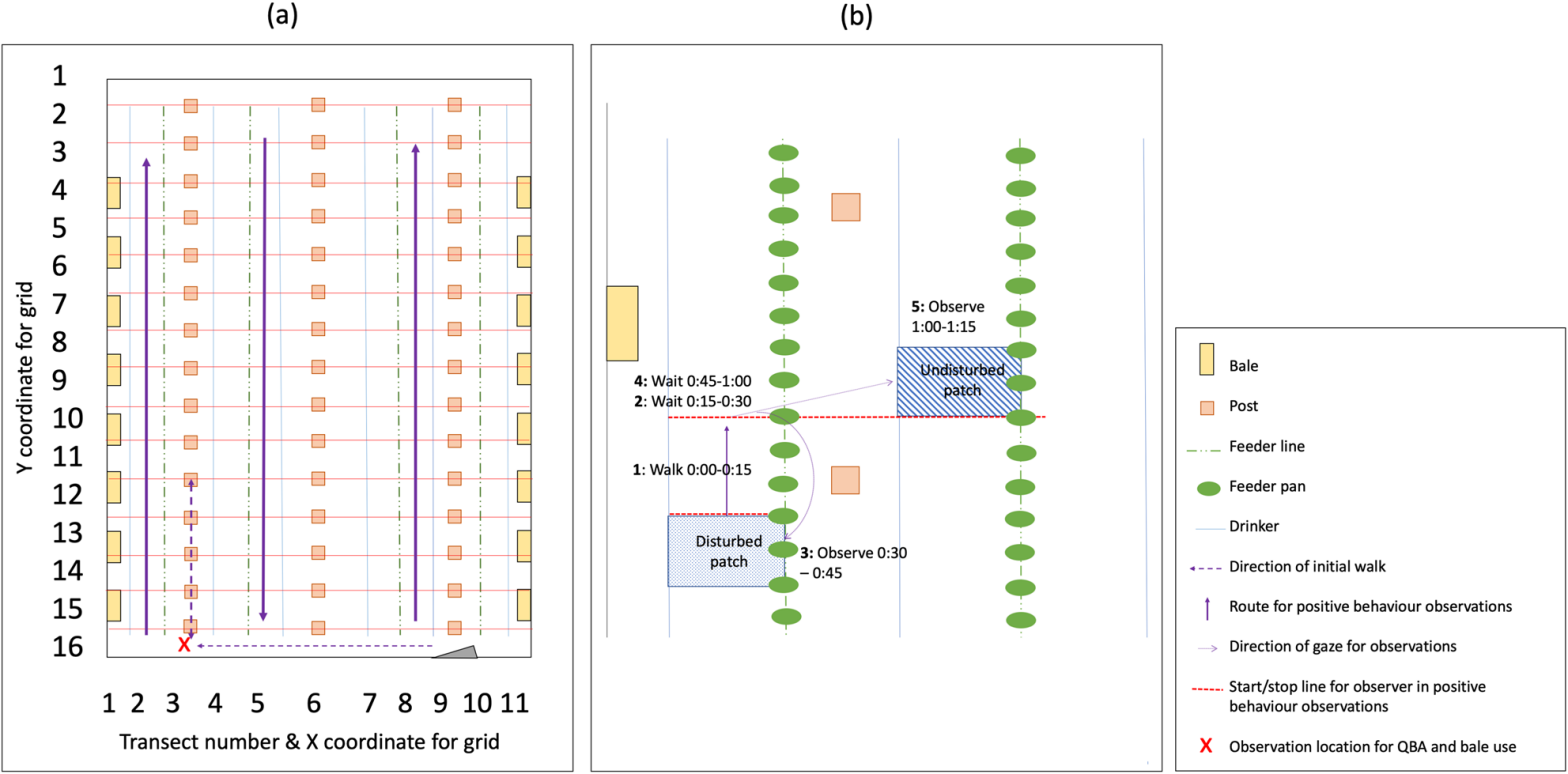
(**a**) Layout of each house, showing theoretical grid for determining random areas of the house (X and Y axis), transect numbers (X axis), initial observation route and location, and Positive behaviour observations route and (**b**) Positive behaviour observations schedule (minutes) and methodology showing Disturbed and Undisturbed patches defined by the edges of the feeder/drinker lines and between 3 feeder pans. Figure created in Microsoft PowerPoint for Mac 2020 Version 16.40 (www.microsoft.com)*.*

The study was conducted on a single farm over four production cycles between January and October, 2018. The farm was managed by the same stockperson throughout. Four houses were allocated as trial houses, all similar in layout, size and orientation. House internal dimensions were 18.1 m × 48.7 m. For Condition 2, Production Cycle 2, insufficient chicks were delivered to the farm such that the house length was reduced to 42.3 m to maintain planned stocking densities. Each house contained six rows of drinkers and four rows of feeders, with 60 feed pans per row. Feeders and drinkers had wires above, installed to discourage birds from perching on them. All houses had three rows of structural posts, and Perspex windows along both long sides. Artificial light was provided by a row of energy-saving compact fluorescent light bulbs down each side of the house and a row of fluorescent tubes down the middle. Each house had two gas heaters, positioned on the same wall, at either end of the house. Fresh wood-shavings litter covered the concrete floor at the start of each production cycle at a depth of 50–70 mm. Small rectangular straw enrichment bales (960 mm × 430 mm × 360 mm) were provided at a rate of 1.5 bales per 1,000 birds (Fig. 7a). Bales remained present in the house throughout the production cycle. Ten 3 m wooden square baton (51 mm × 51 mm) perches at a height of 20 mm were provided per house.

Each condition was managed as per usual commercial practice for that condition. Vaccinations, feed, heating and lighting schedules were implemented according to the breed requirements. Conditions 1–3 received the same diet, whilst Condition 4 received a different diet. Conditions 1–3 had a temperature profile that started at 33 °C and was reduced by 0.4 °C a day to 18 °C whilst Condition 4 started at 34 °C, and was reduced by 0.5 °C a day to 18 °C. All conditions provided natural light from Day 3. By Day 6, Conditions 1–3 provided 6 h of continuous darkness from 21:00, whilst Condition 4 provided a 4 h continuous darkness period from 21:00 followed by a 2 h dark period starting at 03:00 as specified by the breeding company.

Planned maximum stocking density was manipulated by placing different numbers of birds in the house (Table 1). Placement was such as to avoid exceeding maximum stocking densities whilst still accounting for expected mortality as per usual commercial practice.

### Data collection

Stockperson records were used to collate production information and mortality data. Processing welfare outcomes were provided by the processing plant at the end of each production cycle. Additional negative welfare outcomes, and environmental outcomes, were assessed by an observer (A.R.) two days before processing. Positive welfare outcomes were assessed by the same observer at three Production Stages during each production cycle (Days 14 and 28, and 3d before processing) (Table S1). Morphological differences between the breeds meant that it was not possible to blind the observer to treatment.

### Production information

The number of birds remaining in the house was recorded daily by the stockperson. Birds were weighed weekly by the stockperson by penning and weighing 50 random birds. Birds remaining, average weight and internal house dimensions were used to calculate Stocking Density.

### Negative welfare outcomes

#### Mortality

Mortality, and reason, were recorded daily by the stockman. Reason for mortality was defined as culling due to lameness (Leg Culls), culling because moribund or for other reasons (Other Culls) and found dead (Dead). Total Mortality was the sum of these three categories. Values may be subject to some error due to possibility of the stockman not finding all dead birds or those that required culling. Values were expressed as percentages of chicks placed per production cycle.

#### Processing welfare outcomes

Birds from all conditions within a production cycle were processed on the same day and within the same shift, ensuring consistency of catching and processing plant staff and assessors. Dead on Arrival was the percentage of birds that died during transit. Birds were counted prior to being hung on the processing line. Pre-processing Culls included birds deemed to be too small (could fall off the processing line) and emergency culls due to ill-health or sickness. All remaining birds were inspected by poultry meat inspectors. All birds with pathologies were recorded (with reason; Fig. 1) as Post-mortem Inspection Rejections. Data were converted to percentages per production cycle.

#### Additional negative welfare outcomes

Avoidance Distance, Gait Score, Hock Burn and Pododermatitis assessments were undertaken 2d before processing between 09:30 and 14:30. The order of assessing each condition was randomised. Avoidance Distance Tests were conducted first, followed by Gait Scoring, in pre-determined random areas within a theoretical grid of 176 possible areas per house delineated by the posts, and drinker and feeder lines (Fig. 7). In Production Cycles 3 and 4, Hock Burn and Pododermatitis assessments were undertaken in the afternoon of the same day between 14:30 and 16:00, also in pre-determined random areas of the house.

#### Avoidance distance test

Avoidance Distance Tests assessed reactivity of the birds to a person as defined in Welfare Quality^47^. In each of 21 random areas, the observer approached a group of at least 3 birds, slowly squatted for 10 s and then by slowly looking around counted the number of birds within an approximate radius of 1 m (an arm’s length). The mean percentage of birds over the 21 trials was then calculated.

#### Gait score

One hundred birds per condition were scored across 50 random locations. At each location, a 30 cm × 30 cm acetate grid with 5cm^2^ squares numbered 1–36 was held at arm’s length and the two birds closest to a preselected, randomly generated number in the grid were observed to walk for at least 10 steps and scored^48^ using the 6-point Bristol gait scoring method^49^. Zero described a bird with no detectable abnormality and 5 described a bird incapable of walking. Birds scored 4 or 5 were carried to the end of the house to be culled by the stockperson. The data were averaged to obtain a mean gait score per 100 birds.

#### Hock burn and pododermatitis

Roughly 100 birds were penned in four randomly generated locations in each house (Production Cycle 3 and 4 only). All birds penned were assessed. Birds were handled as per routine inspection of hockburn and pododermatitis by the farm manager. Birds were handled according to best practice with two hands around the body (no inversion) allowing for observation of the feet and hocks for scoring^3^. Feet and hocks were scored according to the scales defined in Welfare Quality^47^, whereby 0 equated to no evidence of lesions and 4 to severe lesions. Both legs were inspected and the highest score noted.

### Positive welfare outcomes

Bales Occupied counts, Qualitative Behaviour Assessment (QBA) and Positive behaviour observations were undertaken once at each production stage. For Production Stage 1 and 2 each condition was assessed at either 10:30 or 12:00, in a pseudo-randomised order (two assessments at each time across the four production cycles). At Production Stage 3, each condition was pseudo-randomly assigned to be assessed at 09:30, 11:30, 13:30 or 15:30, due to all conditions requiring assessment on the same day (one assessment at each time across the four production cycles).

The assessments were conducted as follows: The observer entered the house and slowly walked up to the 5th post of Transect 3 to set up a video camera (data not presented). The observer then walked back to the end of Transect 3, near the door-end of the house. After allowing the birds settle for 5 min, they were observed for 10 min and QBA scores were recorded. An instantaneous scan sample of Bales Occupied was then performed. At 20 min after arriving at the end of Transect 3, the observer moved to the starting position for conducting the Positive behaviour observations (Fig. 7).

#### Bales occupied

The number of occupied bales (having one or more birds on top) was counted and expressed as a percentage of the total number of bales in the house.

#### Qualitative behaviour assessment

QBA was undertaken using a fixed list methodology^50^. The list of descriptors included 14 expressive qualities: content, flat, active, playful, flighty, stressed, alert, happy, calm, inquisitive, lethargic, comfortable, lively and relaxed. Following the 10-min observation period, the observer scored the entire flock using a paper-based 0–125 mm Visual Analogue Scale, whereby 0 mm indicated that the expressive quality was absent throughout the whole 10 min and 125 mm indicated the maximum possible expression of the quality. Each term was measured and scored as the distance in mm from 0 to the level marked on the scale.

#### Positive behaviour observations

Transect sampling for positive behaviour was performed using a method modified from Newberry et al.^51^. The feeder and drinker lines were used to delineate 11 transects within each house (Fig. 7a). Transect 11 was on the South-East facing side of each house. Each observation ‘patch’ was defined by the feeder/drinker lines and the distance between 3 feed pans (1.5 m) (Fig. 7b). Scans were made of 54 Undisturbed (U) patches per house, located two transects across from, and ahead of, the observer’s current location, and 57 Disturbed (D) patches through which the observer had slowly walked 75 s previously. For Condition 2, Production Cycle 2, two fewer U and D patches were observed per transect due to the reduced house length (48 U and 51 D patches in total were observed for this house).

To initiate the observation sequence, the observer started a timer and walked slowly to the 3rd feed pan in Transect 2 by 15 s, then waited a further 60 s before walking to the 6th feeder (Step 1) and continuing the cycle of observations (steps 2–5, Fig. 7b). The observer walked slowly up Transect 2 (South-West facing side), observing D patches behind them in Transects 2 and U patches ahead and two transects across in Transect 4 . They then walked slowly down Transect 5, observing Transects 5 (D) and 7 (U) and, finally, up Transect 8, observing Transects 8 (D) and 10 (U) (Fig. 7).

At each patch, the observer recorded the number of birds in a patch that performed each of the following mutually-exclusive behaviours: worm-running, play-fighting, wing-flapping, jumping, running, ground-scratching, vertical wing shaking and perching on wires (Table S2), where the behaviour closest to the first in this list was recorded if the bird performed more than one of these behaviours.

### Environmental outcomes

After completion of Gait Scoring, Litter quality was scored at 6 random locations across the house using the Welfare Quality^47^ classification system. A minimum score of 0 represented “Completely dry and flaky” litter and a maximum score of 4 represented litter that “Sticks to boots once the cap or compacted crust is broken”. The mean of the six scores was calculated to provide one score per house per production cycle.

Ammonia was measured after the Avoidance Distance Test, at five standardised locations throughout each house (two front, two back and one in the middle) using pHydrion paper tests. Each strip was wetted with distilled water and held at bird height for 15 s. The change in colour of the paper indicated ammonia concentration against a colour chart ranging from 0 to 100 ppm. The mean of the 5 scores was calculated to provide one ammonia score per house.

### Statistical analysis

All analyses were undertaken in IBM SPSS version 25 (https://www.ibm.com/analytics/spss-statistics-software).

#### Negative welfare outcomes

Mortality and Processing welfare outcomes were collected only once per production cycle per condition and thus only descriptive data are presented in the text Avoidance Distance, Gait Score, Hock Burn and Pododermatitis all violated the assumptions of a parametric ANOVA and Kruskal–Wallis H tests were utilised. Pairwise comparisons between the four conditions were undertaken using Dunn’s^52^ procedure utilising a Bonferroni correction for multiple comparisons and statistical significance accepted at the p < 0.0083 level.

#### Avoidance distance test

The mean number of birds within arm’s length was compared to a theoretical number of birds within a meter circle (equal to the observer’s arm span) to provide % of birds within arm’s length, where the theoretical number of birds = stocking density (in birds/m2)*π/2. The theoretical number was divided by two, to account for the space taken by the assessor (as per Welfare Quality^47^). Kruskal–Wallis H test and pairwise comparisons were run to explore differences between conditions.

#### Positive welfare outcomes

Bales occupied. Bales Occupied was analysed using a mixed ANOVA with Condition as a fixed effect and Production Stage as a repeated measure. Pairwise comparisons utilising a Bonferroni correction were run to explore differences in Bales Occupied between conditions and production stages.

#### Qualitative behaviour assessment

Scores were analysed using principal component analysis (correlation matrix, no rotation). Separate mixed ANOVAs were run on each identified principal component, with Condition as a fixed effect and Production Stage as a repeated measure. Pairwise comparisons utilising a Bonferroni correction were run to explore differences between conditions and production stages.

#### Positive behaviour observations

Positive behaviours were categorised as Play (including worm-run, play-fight, wing-flap, jump and run), Exploratory (ground-scratch), Comfort (vertical wing shake), Safety (perch on wires) and Any Positive Behaviour (sum of all positive behaviours). The Play category comprised spontaneous, energy-demanding, self-handicapping behaviours performed in a non-life-threatening context^53^. The Exploratory category represented behaviour involved in finding or revealing aspects of the physical environment, Comfort represented body maintenance behaviour (here, measured by vertical wing shakes, a major component of dustbathing behaviour) and Safety represented anti-predator behaviour (here, measuring the skill and agility to perch on narrow, non-stationary, elevated structures).

The expected number of birds in each patch was calculated from the total number of birds in the house on the day of assessment, total area of the house and the proportionate patch size, assuming uniform distribution of birds throughout the house.

Associations of Production Stage, Condition and Disturbance with performance of behaviours in each positive behaviour category were analysed using negative binomial regression with log link function. Scan data were analysed in an ‘events out of trials’ format whereby counts of the number of birds performing behaviours in each behaviour category were summed across all observed Undisturbed and Disturbed patches in the house. By summing the data, any effect of location of patches was accounted for by the similar layout and transect methodology applied to all houses. The offset variable was the log of the sum of the expected number of birds in each observed patch.

Condition 4 was the reference category for comparisons across the four conditions. Maximum planned stocking density associations were explored in Breed B with Condition 3 as the reference category. To explore differences between the two slow-growing breeds (Breed A vs B), Condition 1 was the reference category.

### Ethical statement

This study was carried out with the approval of the University of Bristol Animal Welfare and Ethics Review Body (UB/16/048) and conducted in accordance with UK legislation.

## Supplementary information

Supplementary Information.

## References

[1] European Chicken Commitment https://welfarecommitments.com/europeletter/ (2018).

[2] Better Chicken Commitment, United States and Canada https://welfarecommitments.com/letter.pdf (2019).

[3] Carter E, Hubrecht R (2018). Updated code of practice for the welfare of meat chickens and meat breeding chickens in England. Anim. Welf..

[4] DEFRA (2018). Code of practice for the welfare of meat chickens and meet breeding chickens.

[5] Buijs S, Keeling L, Tuyttens F (2011). Using motivation to feed as a way to assess the importance of space for broiler chickens. Anim. Behav..

[6] Febrer K, Jones TA, Donnelly CA, Dawkins MS (2006). Forced to crowd or choosing to cluster? Spatial distribution indicates social attraction in broiler chickens. Anim. Behav..

[7] Buijs S (2010). Resting or hiding? Why broiler chickens stay near walls and how density affects this. Appl. Anim. Behav. Sci..

[8] Dawkins MS, Donnelly CA, Jones TA (2004). Chicken welfare is influenced more by housing conditions than stocking density. Nature.

[9] Hall AL (2001). The effect of stocking density on the welfare and behaviour of broiler chickens reared commercially. Anim. Welf..

[10] Knowles TG (2008). Leg disorders in broiler chickens: prevalence, risk factors and prevention. PLoS ONE.

[11] Bailie CL, Ijichi C, O’Connell NE (2018). Effects of stocking density and string provision on welfare-related measures in commercial broiler chickens in windowed houses. Poult. Sci..

[12] BenSassi N (2019). On-farm broiler chicken welfare assessment using transect sampling reflects environmental inputs and production outcomes. PLoS ONE.

[13] Arnould C, Faure JM (2004). Use of pen space and activity of broiler chickens reared at two different densities. Appl. Anim. Behav. Sci..

[14] Dozier WA (2005). Stocking density effects on growth performance and processing yields of heavy broilers. Poult. Sci..

[15] Davies, J. *Slow-growing birds are fast becoming mainstream*. Poultry World. https://www.poultryworld.net/Meat/Articles/2019/7/Slow-growing-birds-are-fast-becoming-mainstream-454287E/ (2019).

[16] Bokkers EA, Koene P (2003). Behaviour of fast-and slow growing broilers to 12 weeks of age and the physical consequences. Appl. Anim. Behav. Sci..

[17] Castellini C (2016). Adaptation to organic rearing system of eight different chicken genotypes: behaviour, welfare and performance. Ital. J. Anim. Sci..

[18] Dixon LM (2020). Slow and steady wins the race: the behaviour and welfare of commercial faster growing broiler breeds compared to a commercial slower growing breed. PLoS ONE.

[19] Kestin SC, Su G, Sørensen P (2001). Relationships in broiler chickens between lameness, liveweight, growth rate and age. Vet. Rec..

[20] Corr SA, Gentle MJ, McCorquodale CC, Bennett D (2003). The effect of morphology on walking ability in the modern broiler: a gait analysis study. Anim. Welf..

[21] Bokkers E, Koene P (2004). Motivation and ability to walk for a food reward in fast- and slow-growing broilers to 12 weeks of age. Behav. Proc..

[22] Lawrence AB, Vigors B, Sandøe P (2019). What is so positive about positive animal welfare? A critical review of the literature. Animals.

[23] Mellor DJ (2016). Updating animal welfare thinking: moving beyond the “Five Freedoms” towards “A Life Worth Living”. Animals.

[24] FAWC (2009). Farm Animal Welfare in Great Britain Past Present and Future.

[25] Edgar JL, Mullan SM, Pritchard JC, McFarlane UJC, Main DCJ (2013). Towards a ‘Good Life’ for farm animals: development of a resource tier framework to achieve positive welfare for laying hens. Animals.

[26] Van der Most P, de Jong HB, Parmentier H, Verhulst S (2011). Trade-off between growth and immune function: a meta-analysis of selection experiments. Funct. Ecol..

[27] Vasdal G, Moe R, De Jong I, Granquist E (2018). The relationship between measures of fear of humans and lameness in broiler chicken flocks. Animal.

[28] Tuyttens FAM (2015). Assessment of welfare of Brazilian and Belgian broiler flocks using the Welfare Quality protocol. Poult. Sci..

[29] Danbury TC, Weeks CA, Waterman-Pearson AE, Kestin SC, Chambers JP (2000). Self-selection of the analgesic drug carprofen by lame broiler chickens. Vet. Rec..

[30] McGeown D, Danbury TC, Waterman-Pearson AE, Kestin SC (1999). Effect of carprofen on lameness in broiler chickens. Vet. Rec..

[31] Vestergaad S, Sanotra GS (1999). Relationships between leg disorders and changes in the behaviour of broiler chickens. Vet. Rec..

[32] Weeks CA, Danbury TD, Davies HC, Hunt P, Kestin SC (2000). The behaviour of broiler chickens and its modification by lameness. Appl. Anim. Behav. Sci..

[33] Hester PY (1994). The role of environment and management on leg abnormalities in meat-type fowl. Poult. Sci..

[34] Haslam SM (2007). Factors affecting the prevalence of footpad dermatitis, hock burn and breast burn in broiler chicken. Br. Poult. Sci..

[35] Shepherd EM, Fairchild BD (2010). Footpad dermatitis in poultry. Poult. Sci..

[36] Bassler AW (2013). Potential risk factors associated with contact dermatitis, lameness, negative emotional state, and fear of humans in broiler chicken flocks. Poult. Sci..

[37] Muri K, Stubsjøen S, Vasdal G, Moe RO, Granquist EG (2019). Associations between qualitative behaviour assessments and measures of leg health, fear and mortality in Norwegian broiler chicken flocks. Appl. Anim. Behav. Sci..

[38] Bizeray D, Estevez I, Leterrier C, Faure J (2002). Effects of increasing environmental complexity on the physical activity of broiler chickens. Appl. Anim. Behav. Sci..

[39] Duncan IJH (1988). Behaviour and behavioural needs. Poult. Sci..

[40] Duncan IJH, Hughes BO (1972). Free and operant feeding in domestic fowls. Anim. Behav..

[41] Vasdal G, Vas J, Newberry RC, Moe RO (2019). Effects of environmental enrichment on activity and lameness in commercial broiler production. J. Appl. Anim. Welf. Sci..

[42] Held SDE, Špinka M (2011). Animal play and animal welfare. Anim. Behav..

[43] Baxter M, Bailie C, O’Connell N (2019). Play behaviour, fear responses and activity levels in commercial broiler chickens provided with preferred environmental enrichments. Animal.

[44] Naseem S, King AJ (2018). Ammonia production in poultry houses can affect health of humans, birds, and the environment—techniques for its reduction during poultry production. Environ. Sci. Pollut. Res..

[45] Red Tractor. *Chicken Standards: Broilers and Poussin* (updated 1st October 2019), Version 4.2. https://assurance.redtractor.org.uk/contentfiles/Farmers-6803.pdf (2019).

[46] Shields SJ, Garner JP, Mench JA (2004). Dustbathing by broiler chickens: a comparison of preference for four different substrates. Appl. Anim. Behav. Sci..

[47] Welfare Quality® (2009). assessment Protocol for Poultry (Broilers, Laying Hens).

[48] Kells A, Dawkins MS, Borja MC (2001). The effect of a ‘freedom food’ enrichment on the behaviour of broilers on commercial farms. Anim. Welf..

[49] Kestin SC, Knowles TG, Tinch AE, Gregory NG (1992). Prevalence of leg weakness in broiler chickens and its relationship with genotype. Vet. Rec..

[50] Clarke T, Pluske J, Fleming P (2016). Are observer ratings influenced by prescription? A comparison of Free Choice Profiling and Fixed List methods of Qualitative Behavioural Assessment. Appl. Anim. Behav. Sci..

[51] Newberry, R. C. et al. Chickens play in the wake of humans. In *Proceedings of the 52nd Congress of the International Society for Applied Ethology*, 199 (Wageningen Academic Publishers, Wageningen, 2018).

[52] Dunn OJ (1964). Multiple comparisons using rank sums. Technometrics.

[53] Spinka M, Newberry RC, Bekoff M (2001). Mammalian play: training for the unexpected. Q. Rev. Biol..

