# Supplementary material for: Slow-growing broilers are healthier and express more behavioural indicators of positive welfare
Source: Sci Rep. 2020 Sep 16;10:15151. doi: 10.1038/s41598-020-72198-x (PMC7494998; doi:10.1038/s41598-020-72198-x)
Supplement: Supplementary file 1 — Supplementary Information. [file 41598_2020_72198_MOESM1_ESM.docx]

**Slow-growing broilers are healthier and express more behavioural indicators of positive welfare**

*Ann C Rayner*****^1,2^****, Ruth C. Newberry****^3^****, Judit Vas****^3^****, Siobhan Mullan****^2^***

**^1^**FAI Farms, Northfield Farm, Wytham OX2 8QJ, United Kingdom,

**^2^**University of Bristol, Bristol Veterinary School, Division of Food Animal Science, Langford BS40 5DU, United Kingdom,

**^3^**Norwegian University of Life Sciences, Faculty of Biosciences, Department of Animal and Aquacultural Sciences, 1432 Ås, Norway

[annie.rayner@faifarms.com](mailto:annie.rayner@faifarms.com)

## Supplementary Information

*Table S1: Measures and time points when assessed in each Condition*

| **Assessment category** | **Data source** | **Timing of measurement** | **Measure** |
| --- | --- | --- | --- |
| Production information | Stockperson records | Daily | Remaining birds / Stocking density |
|  |  | Weekly | Weight / Growth rate |
| Negative welfare outcomes | Stockperson records | Daily | Mortality (+ reason) |
|  | Processor | At processing | Dead on Arrival |
|  |  |  | Pre-processing Culls |
|  |  |  | Total Post-Mortem Inspection Rejections (+ reason) |
|  | Observer (AR) | Two days before processing | Gait Score |
|  |  |  | Hock Burn (Production Cycles 3 and 4 only) |
|  |  |  | Pododermatitis (Production Cycles 3 and 4 only) |
|  |  |  | Avoidance Distance Test |
| Positive welfare outcomes | Observer (AR) | 3 Production Stages (Days 14 and 28, and 3 days before processing) | Bales Occupied |
|  |  |  | Qualitative Behaviour Assessment (QBA) |
|  |  |  | Positive Behaviour Observations |
| Environmental outcomes | Observer (AR) | Two days before processing | Litter Quality |
|  |  |  | Ammonia |

*Table S2: Ethogram of mutually-exclusive behaviours recorded when birds were undisturbed, or 75 s following a disturbance (adapted from Newberry et al. [51])*

| Behaviour category | Behaviour | Description |
| --- | --- | --- |
| Play* | Worm-run | While walking or running excitedly, bird carries a small object projecting from the beak, such as a large piece of wood shaving or peat, piece of paper or plastic, or a feather. The bird makes rapid changes of direction and normally attracts other birds to follow. |
|  | Play-fight | Bird runs or jumps directly towards the head of another standing bird which may simultaneously rapidly approach. If both birds face each other and stare while in close proximity, both birds are counted. If one bird approaches and stares at another standing bird which does not respond, only that bird is counted. If playful movements and sudden stops are not directed to any particular bird, not recorded as play fight. |
|  | Wing-flap | While active and not lying down, bird raises and rapidly lowers both wings simultaneously, usually several times in rapid succession. Usually occurs when running or jumping, aiding in propelling the body. Does not include slow stretching of wings, holding wings out but not flapping, or body shaking. |
|  | Jump | While active and not lying down, bird moves both feet off substrate simultaneously; may jump up off the floor (e.g. up into the air) |
|  | Run | While active and not lying down, bird takes at least three rapid steps forward with one foot after the other, with both feet briefly lifted off the substrate during strides, resulting in moving the body rapidly from one location to another. |
| Exploration | Ground-scratch | While active and not lying down, bird rakes the substrate with the toes and claws using a rapid backward kicking movement of the leg and foot. |
| Comfort | Vertical wing shake | While dustbathing on the ground in a recumbent position with fluffed feathers, bird simultaneously and rapidly lifts the wings up and down multiple times, typically accompanied with vigorous leg movements; wings are held close to the body, scooping loose substrate material up into the feathers. Does not include scratching with feet while lying down when unaccompanied by wing movements. |
| Safety | Perch on wire | Bird sits or stands on wire above drinker or feeder lines (wooden perches and bales were not present in the observed patches). |

* Behaviours are listed in order of precedence for determining which behaviour to record when a bird was performing multiple behaviours in the same scan.


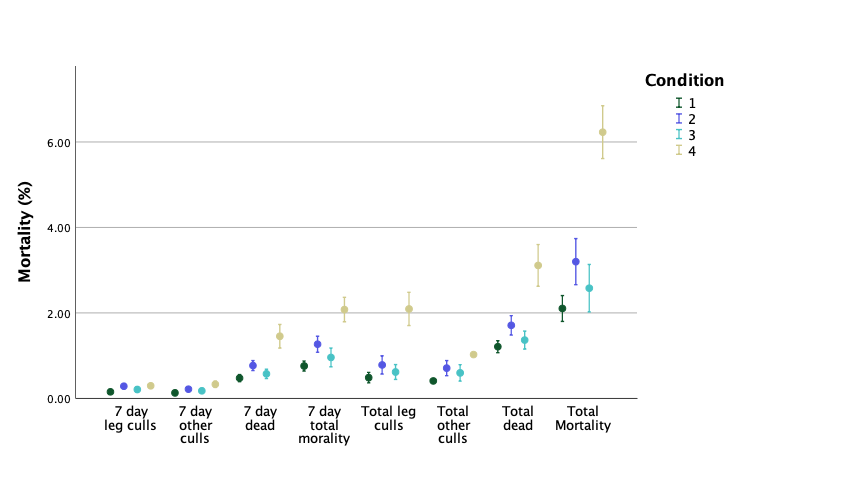


*Figure S1: Mean (± SE) 7d and Total Mortality % (+ reason) across each Condition (n=4 for Conditions 2,3 and 4; n=3 for Condition 1).*


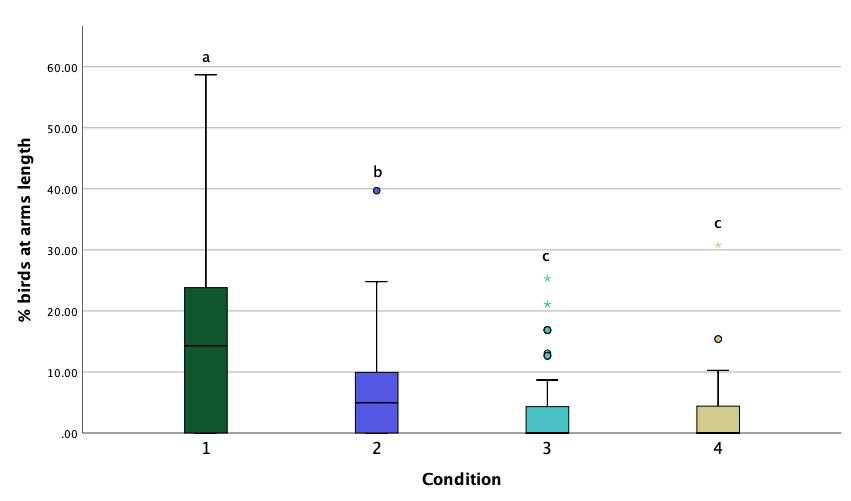


*Figure S2: Median (with IQR) % of birds within one meter of the observer during the Avoidance Distance Test. Whiskers show 95% of range, dots show outliers and stars show extreme outliers (>3 times the height of the box) (n=84 per Condition). Different letters indicate differences in % distribution between Conditions identified by pairwise comparisons using Dunn’s [52] procedure (p<0.0083).*

*Table S3: Qualitative Behaviour Assessment descriptor loadings onto principal components PC1 and PC2. Loadings higher than 0.60 for each term are bold typed.*

| PC1 | | PC2 | |
| --- | --- | --- | --- |
| **Happy** | **0.819** | **Calm** | **0.714** |
| **Active** | **0.782** | Happy | 0.271 |
| **Playful** | **0.756** | Content | 0.269 |
| **Content** | **0.734** | Relaxed | 0.256 |
| **Lively** | **0.671** | Flat | 0.200 |
| **Relaxed** | **0.670** | Stressed | 0.029 |
| Comfortable | 0.586 | Inquisitive | -0.015 |
| Inquisitive | 0.552 | Lethargic | -0.083 |
| Calm | 0.425 | Playful | -0.221 |
| Alert | 0.188 | Comfortable | -0.249 |
| Flighty | -0.124 | Active | -0.298 |
| **Lethargic** | **-0.606** | Lively | -0.300 |
| **Stressed** | **-0.626** | **Alert** | **-0.639** |
| **Flat** | **-0.744** | **Flighty** | **-0.789** |

*Table S4: Play behaviour (including worm-run, play-fight, wing-flap, jump, run), Exploratory behaviour (ground-scratching) and Comfort behaviour (vertical wing shaking) and Any Positive Behaviour (all categories summed), in Undisturbed and Disturbed patches.*

| Explanatory variable | Wald Chi-square | Df | P | Variable levels | p | Exp(β) | 95% Wald Confidence Interval for Exp(β) | |
| --- | --- | --- | --- | --- | --- | --- | --- | --- |
|  |  |  |  |  |  |  | lower | upper |
| Play behaviours – Undisturbed | | | | | | | | |
| Production Stage | 15.96 | 2 | <0.001 | Production Stage 1 | <0.001 | 4.48 | 2.15 | 9.36 |
|  |  |  |  | Production Stage 2 | 0.039 | 2.19 | 1.04 | 4.61 |
|  |  |  |  | Production Stage 3 | - | 1.00 | - | - |
| Condition | 3.72 | 3 | 0.293 | Condition 1 | 0.207 | 1.74 | 0.74 | 4.09 |
|  |  |  |  | Condition 2 | 0.093 | 2.06 | 0.89 | 4.80 |
|  |  |  |  | Condition 3 | 0.095 | 2.06 | 0.88 | 4.83 |
|  |  |  |  | Condition 4 | - | 1.00 | - | - |
| Play behaviours – Disturbed | | | | | | | | |
| Production Stage | 42.69 | 2 | <0.001 | Production Stage 1 | <0.001 | 0.12 | 0.06 | 0.25 |
|  |  |  |  | Production Stage 2 | 0.982 | 0.99 | 0.49 | 2.00 |
|  |  |  |  | Production Stage 3 | . | 1.00 | . | . |
| Condition | 13.46 | 3 | 0.004 | Condition 1 | 0.001 | 4.09 | 1.79 | 9.34 |
|  |  |  |  | Condition 2 | 0.006 | 3.20 | 1.40 | 7.29 |
|  |  |  |  | Condition 3 | 0.005 | 3.27 | 1.43 | 7.48 |
|  |  |  |  | Condition 4 | . | 1.00 | . | . |
| Exploratory behaviour – Undisturbed | | | | | | | | |
| Production Stage | 25.23 | 2 | <0.001 | Production Stage 1 | <0.001 | 15.88 | 5.17 | 48.74 |
|  |  |  |  | Production Stage 2 | 0.011 | 4.56 | 1.41 | 14.72 |
|  |  |  |  | Production Stage 3 | . | 1.00 | . | . |
| Condition | 12.57 | 3 | 0.006 | Condition 1 | 0.008 | 5.95 | 1.60 | 22.09 |
|  |  |  |  | Condition 2 | <0.001 | 10.16 | 2.81 | 36.76 |
|  |  |  |  | Condition 3 | 0.009 | 5.64 | 1.53 | 20.74 |
|  |  |  |  | Condition 4 | . | 1.00 | . | . |
| Exploratory behaviour – Disturbed | | | | | | | | |
| Production Stage | 1.52 | 2 | 0.469 | Production Stage 1 | 0.544 | 1.56 | 0.37 | 6.62 |
|  |  |  |  | Production Stage 2 | 0.224 | 2.36 | 0.59 | 9.39 |
|  |  |  |  | Production Stage 3 | . | 1.00 | . | . |
| Condition | 3.83 | 3 | 0.280 | Condition 1 | 0.091 | 3.77 | 0.81 | 17.59 |
|  |  |  |  | Condition 2 | 0.646 | 1.50 | 0.27 | 8.32 |
|  |  |  |  | Condition 3 | 0.683 | 1.43 | 0.26 | 8.01 |
|  |  |  |  | Condition 4 | . | 1.00 | . | . |
| Comfort behaviour– Undisturbed | | | | | | | | |
| Production Stage | 9.077 | 2 | 0.011 | Production Stage 1 | 0.010 | 6.45 | 1.55 | 26.83 |
|  |  |  |  | Production Stage 2 | 0.003 | 8.67 | 2.10 | 35.81 |
|  |  |  |  | Production Stage 3 | . | 1 | . | . |
| Condition | 6.264 | 3 | 0.099 | Condition 1 | 0.020 | 4.82 | 1.29 | 18.10 |
|  |  |  |  | Condition 2 | 0.056 | 3.75 | 0.97 | 14.54 |
|  |  |  |  | Condition 3 | 0.328 | 2.09 | 0.48 | 9.12 |
|  |  |  |  | Condition 4 | . | 1 | . | . |
| Any Positive behaviour – Undisturbed | | | | | | | | |
| Production Stage | 14.26 | 2 | 0.001 | Production Stage 1 | <0.001 | 4.05 | 1.96 | 8.39 |
|  |  |  |  | Production Stage 2 | 0.073 | 1.96 | 0.94 | 4.08 |
|  |  |  |  | Production Stage 3 | - | 1.00 | - | - |
| Condition | 6.75 | 3 | 0.080 | Condition 1 | 0.053 | 2.30 | 1.00 | 5.30 |
|  |  |  |  | Condition 2 | 0.024 | 2.62 | 1.13 | 6.10 |
|  |  |  |  | Condition 3 | 0.030 | 2.54 | 1.09 | 5.90 |
|  |  |  |  | Condition 4 | - | 1.00 | - | - |
| Any positive behaviour – Disturbed | | | | | | | | |
| Production Stage | 41.63 | 2 | <0.001 | Production Stage 1 | <0.001 | 0.13 | 0.06 | 0.26 |
|  |  |  |  | Production Stage 2 | 0.982 | 0.99 | 0.49 | 2.00 |
|  |  |  |  | Production Stage 3 | - | 1.00 | - | - |
| Condition | 13.77 | 3 | 0.003 | Condition 1 | <0.001 | 4.17 | 1.82 | 9.51 |
|  |  |  |  | Condition 2 | 0.005 | 3.24 | 1.42 | 7.37 |
|  |  |  |  | Condition 3 | 0.005 | 3.28 | 1.44 | 7.50 |
|  |  |  |  | Condition 4 | - | 1.00 | - | - |


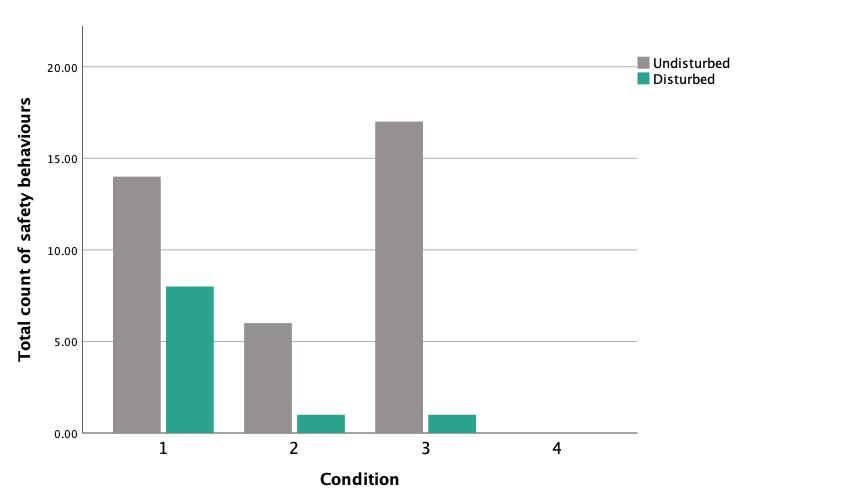


*Figure S3: Safety behaviour (perching) counts by Condition, summed across three Production Stages and four Production Cycles*
